# Cross-Language Influences in the Processing of Multiword Expressions: From a First Language to Second and Back

**DOI:** 10.3389/fpsyg.2021.666520

**Published:** 2021-06-24

**Authors:** Lingli Du, Irina Elgort, Anna Siyanova-Chanturia

**Affiliations:** ^1^School of Foreign Languages, Henan University of Technology, Zhengzhou, China; ^2^School of Linguistics and Applied Language Studies, Te Herenga Waka – Victoria University of Wellington, Wellington, New Zealand; ^3^College of Foreign Languages, Ocean University of China, Qingdao, China

**Keywords:** multiword expressions, binomials, cross-language influence, congruency, frequency, English, Chinese, priming

## Abstract

The present study investigated cross-language influences in the processing of binomial expressions (*knife and fork*), from a first language (L1) to a second language (L2) and from L2 to L1. Two groups of unbalanced bilinguals (Chinese/L1-English/L2 and English/L1-Chinese/L2) and a control group of English monolinguals performed a visual lexical decision task that incorporated unmasked priming. To assess cross-language influences, we used three types of expressions: congruent binomials (English binomials that have translation equivalents in Chinese), English-only binomials, and Chinese-only binomials translated into English. Lexical decision latencies to the last word (*fork*) in a binomial (*knife and fork*) were compared with response latencies to the same word in a matched control phrase (*spoon and fork*). We found that (1) Chinese-English bilinguals showed a significant priming effect for congruent binomials but no facilitation for English-only binomials, (2) English–Chinese bilinguals showed a trend toward priming for congruent binomials, which did not reach statistical significance, and no priming for English-only binomials, (3) English monolinguals showed comparable priming for congruent and English-only binomials. With respect to the Chinese-only binomials, none of the three participant groups showed priming for translated Chinese-only binomials over controls. These findings suggest that L1 influences the processing of L2 binomials, and that there may be some cross-linguistic influence in the opposite direction, i.e., from L2 to L1, although to a lesser extent.

## Introduction

Research in bilingual language processing extends beyond single words, to lexical units known as multiword expressions (MWEs), such as idioms (*kick the bucket*) and collocations (*strong tea*). Bilingual research shows that a bilingual’s first language (L1) can influence the processing of a second language (e.g., Keatley et al., 1994; Kim and Davis, 2003; Duyck, 2005; Schoonbaert et al., 2007), and that a non-dominant second language (L2) can also influence the processing in the dominant L1 (Jiang, 1999; van Hell and Dijkstra, 2002; Schoonbaert et al., 2009). Specifically, bilingual processing has been found to entail cross-language activation (e.g., Kroll and Bialystok, 2013; Conklin, 2020; Whitford and Titone, 2019). In bilingual processing beyond the word level, L1 has been found to influence the processing of MWEs in an L2 (Wolter and Gyllstad, 2011, 2013; Carrol and Conklin, 2014, 2017; Carrol et al., 2016), but cross-linguistic influences in the L2-L1 direction are less clear. The present article examines whether such bidirectional cross-linguistic influences (CLIs) exist at the phrase level.

Multiword expressions are heterogeneous, consisting of a large set of expression types, such as idioms (*kick the bucket*), lexical bundles (*in the middle of*), binomials (*bride and groom*), collocations (*strong tea*), and other phrasal elements (Siyanova-Chanturia and van Lancker Sidtis, 2019). MWEs vary greatly in frequency of occurrence^1^. However, what they have in common is that they are highly familiar and predictable to a native speaker (Siyanova-Chanturia and Martinez, 2015). For example, on hearing or reading the beginning of *fish and* …, a proficient language user is likely to complete it with the most likely word(s) *chips*. Due to their frequency and predictability, MWEs are processed faster than matched novel strings of language by L1 speakers (Arnon and Snider, 2010; Tremblay et al., 2011; Vilkaite, 2016) and L2 speakers (Jiang and Nekrasova, 2007; Siyanova-Chanturia et al., 2011; Hernández et al., 2016). Specifically, using priming paradigms, studies have found that the beginning of a MWE can prime its terminal word (Durrant and Doherty, 2010; Wolter and Gyllstad, 2011; Carrol and Conklin, 2014). For instance, in a primed lexical decision task, Wolter and Gyllstad (2011) observed *collocational* priming among L1 and L2 speakers for prime-target item pairs consisting of verb-noun collocations (*find job*) when compared with unrelated item pairs (*hear part*). Similarly, in an eye-tracking study, Carrol et al. (2016) found *idiom* priming effects in L1 and L2 reading, such that the final words in idioms (*spill the beans*) were skipped more often than the last words in control phrases (*drop the beans*).

### Evidence for Congruency Effect in MWE Processing in an L2

There has been growing interest in how congruency (i.e., similarity in form and meaning between the L1 and L2) may affect MWE processing in *bilinguals*. An L2 expression is congruent if it has a word-for-word translation equivalent form in the L1 (Conklin and Carrol, 2018). Cross-language overlap plays an important role in the L2 MWE processing; L2 speakers can process congruent MWEs more rapidly than incongruent L2-only MWEs (Yamashita and Jiang, 2010; Wolter and Gyllstad, 2011, 2013; Carrol et al., 2016). Using a phrase-acceptability judgment task, Yamashita and Jiang (2010) found that lower proficiency Japanese English as a foreign language (EFL) learners made more errors with and responded more slowly to incongruent English-only (verb-noun and adjective-noun) collocations than to congruent collocations. Higher proficiency Japanese English as a second language (ESL) users also made more errors on incongruent collocations than on congruent ones, but they responded equally fast to the two types of collocations, indicating that proficiency may partially offset the effect of congruency. However, Wolter and Gyllstad (2011, 2013) and Wolter and Yamashita (2018) observed that even high proficiency L2 speakers showed a robust processing advantage in response times for congruent vs. incongruent collocations. They observed that congruent collocations (verb-noun and adjective-noun) were processed significantly faster and more accurately than incongruent (English-only) collocations by advanced Swedish learners of English, in lexical decision (Wolter and Gyllstad, 2011) and acceptability judgment experiments (Wolter and Gyllstad, 2013). Comparable results were reported with Japanese-English bilinguals (Wolter and Yamashita, 2018). Similarly, L2 idiom processing studies also found a facilitative effect of congruency (Titone et al., 2015; Carrol et al., 2016). For example, in an eye-tracking study, Carrol et al. (2016) found that advanced Swedish learners of English processed congruent idioms faster than literal controls, whereas they processed incongruent (English-only) idioms and literal controls in a similar way (as indexed by the likelihood of skipping of the final word). Together, these studies suggest that congruency between languages can facilitate L2 MWE processing.

Although the above studies show a clear influence of L1 knowledge on the processing of L2 MWEs, evidence is mixed with regard to whether *unfamiliar* translated L1-only MWEs that do not exist in the L2 can also show a processing advantage over matched controls. L2 idiom studies have found that the L1 influence extends to the processing of translated L1-only items. Carrol and Conklin (2014), for example, found that high-proficiency Chinese–English bilinguals showed priming in a lexical decision task for translated Chinese-only idioms (e.g., *draw a snake and add* … *feet*) relative to matched controls (e.g., *draw a snake and add* … *hair*), whereas a control group of English monolinguals showed no priming. Similar findings were reported in a follow-up eye-tracking study with a similar population (Carrol and Conklin, 2017). Furthermore, in an eye-tracking study with highly proficient Swedish-English bilinguals, Carrol et al. (2016) directly compared facilitation for congruent idioms (e.g., *lose your head*) and Swedish-only idioms (e.g., *play monkey*) relative to literal controls (e.g., *hurt your head*, *taste monkey*, respectively). They found that translated Swedish-only idioms showed the same level of facilitation as did congruent idioms, and that there was no additional facilitatory effect for congruent idioms due to additional experience of the same combinations in the L2. These results suggest that the familiarity with L1 MWEs is a key driver of L2 idiom processing advantage, above and beyond L2 experience.

Conversely, studies on L2 *collocational* processing did not report a processing advantage for translated L1-only collocations compared to matched controls. In a study by Wolter and Yamashita (2015), two groups of Japanese-English bilinguals (intermediate and advanced) and English monolinguals completed a double lexical decision task, where they decided whether or not both words of a collocation, presented simultaneously, were real English words. Three types of items were used: translated Japanese-only collocations (*high effect*), English-only collocations (*busy road*), and non-collocations (*bad gift*). They found no processing advantage for translated Japanese-only collocations over non-collocations in either group of Japanese–English bilinguals, suggesting no activation of known L1 collocations in the processing of the translated L1-only items. In a follow-up study that encouraged focus on meaning rather than form, intermediate and advanced Japanese–English bilinguals and English monolinguals performed an acceptability judgment task, in which they decided as quickly as possible whether or not a two-word combination (*thick fog*) was commonly used in English (Wolter and Yamashita, 2018). Again, the results showed no significant processing advantage for translated Japanese-only collocations (*weak rain*) over non-collocations (*proud idea*).

### Mechanisms Behind Congruency Effect in MWE Processing in an L2

The issue of whether or not bilinguals show an advantage in the processing of L1-only MWEs translated into the L2 has important implications for understanding the mechanisms behind the congruency effect in the processing of L2 idioms and collocations and other MWEs (Wolter and Gyllstad, 2011, 2013; Carrol and Conklin, 2014, 2017; Yamashita, 2018). The L1 influence on L2 MWE processing may be explained in two ways. One explanation attributes the observed L1-on-L2 effect to the online activation of known L1 MWEs, i.e., the L1 MWE activation account (see also Yamashita, 2018; Zeng et al., 2020). The second explanation attributes the congruency advantage to the age of acquisition effect, assuming that congruent MWEs are acquired earlier and faster than L2-only MWEs. We will refer to this as the L2 MWE experience account.

In the L1 MWE activation account, known L1 MWEs are assumed to be automatically activated in L2 processing, leading to their faster processing (e.g., Wolter and Gyllstad, 2011; Carrol and Conklin, 2014; Carrol et al., 2016). For example, Carrol and Conklin (2014, concerning idioms) proposed that L2 words automatically activate L1 equivalents in bilinguals, which, in turn, trigger a known L1 sequence via direct retrieval of a unitary form. Likewise, concerning collocations, Wolter and Gyllstad (2011) proposed that an L2 word activates not only its L2 collocates (e.g., *strong* activates its collocate *tea*), but also its L1 translation equivalent (*strong* – *nong*/*浓*), which in turn activates its L1 collocates via collocational priming (*cha*/*茶* – *tea*). A number of studies have shown that when bilinguals process language in their L2, they obligatorily activate the L1 translation equivalents (i.e., cross-language translation priming: e.g., the L2 word *horse* primes its L1 translation equivalent *ma*/*马*) (Wu and Thierry, 2010; Zhang et al., 2011; Wu et al., 2013). For instance, in a relatedness judgment task with Chinese-English bilinguals, Wu and Thierry (2010) found the N400 effect for English word pairs whose Chinese translations had a repeated phonological component, e.g., *experience* [***Jing*** *Yan经验*]-*surprise* [***Jing*** *Ya惊讶*]. They concluded that L1 translations are automatically activated in L2 processing. Thus, it is plausible that due to cross-language activation in bilinguals, cross-language priming may extend to the phrase level. Under the L1 MWE activation account, when translation equivalents of L1 MWEs are first encountered in an L2, some facilitatory L1 influence in their processing should be observed (Carrol et al., 2016). This is supported by empirical studies that have reported on idiom priming effects for L1-only idioms over literal controls in bilinguals when encountered in the L2 for the first time (Carrol and Conklin, 2014, 2017; Carrol et al., 2016). However, no support is found in the processing of translated L1-only *collocations* (Wolter and Yamashita, 2015, 2018).

In the L2 MWE experience account, no assumption is made about automatic activation of L1 MWE translation equivalents in L2 processing and, therefore, no priming for translated L1-only MWEs is predicted in L2 processing tasks. According to Wolter and colleagues (e.g., Wolter and Gyllstad, 2013; Wolter and Yamashita, 2015, 2018), congruent MWEs are acquired before incongruent MWEs due to positive L1 transfer, and, thus, congruent MWEs should be processed faster than incongruent MWEs. This would be analogous to the age-of-acquisition (AoA) effect, i.e., words that are acquired earlier are processed faster than words that are acquired later (Morrison and Ellis, 1995; Ellis and Morrison, 1998; Juhasz and Rayner, 2006). Multiple studies have shown that L1 plays an important role in the *acquisition* of L2 MWEs (Nesselhauf, 2005; Römer et al., 2014; Sonbul et al., 2020). For instance, Yamashita and Jiang (2010) found that both lower- and higher-proficiency Japanese-English bilinguals, but not monolingual English controls, made fewer errors on congruent collocations than incongruent L2-only collocations in a phrase-acceptability judgment task. They concluded that acquiring congruent L2 collocations takes less time and requires less exposure to the L2 than incongruent L2-only collocations. As Yamashita and Jiang (2010) posited, a congruent L2 MWE and its L1 counterpart share the identical or very similar concept, and thus bilinguals can easily accept and store congruent MWEs in memory by simply resorting to L1 expressions (Yamashita and Jiang, 2010, p. 662). Thus, it is plausible that congruent MWEs are acquired earlier than incongruent, L2-only MWEs.

With respect to the second claim by Wolter and colleagues (Wolter and Gyllstad, 2013; Wolter and Yamashita, 2015, 2018) that earlier acquired MWEs are processed faster than later acquired MWEs, there is empirical evidence showing that AoA affects the processing of units longer than a word. Using a phrasal decision task, Arnon et al. (2017) found that adults responded faster to early acquired phrases (*for the baby*) compared to late-acquired phrases (*for the teacher*), suggesting the AoA effect for units beyond single word level. Under this L2 MWE experience account, translated L1-only items which are encountered in the L2 for the first time should not show a processing advantage over matched controls in bilinguals. This view has found support in the results from Wolter and Yamashita (2015, 2018). Taken together, the L1 MWE activation account and L2 MWE experience account make differential predictions about the processing of translated L1-only MWEs when encountered in the L2 for the first time, although evidence is still mixed. Further research is needed to explore the processing of translated L1-only MWEs in bilinguals.

### L2 Influence on Lexical Processing in an L1

Additionally, although the reviewed studies have established that the L1 knowledge influences the processing of L2 MWEs, whether the processing of L1 MWEs is affected by the knowledge of L2 has not been sufficiently addressed in the literature. This issue, however, has been investigated in lexical, single word, processing research. The literature on the topic suggests that even weak, non-dominant L2 may affect the processing of words in the dominant L1 (van Hell and Dijkstra, 2002; Schoonbaert et al., 2009; Degani et al., 2011). For instance, van Hell and Dijkstra (2002) found that L1 words that are cognates with their L2 translations (e.g., Dutch–English: *bakker-baker*) lead to faster lexical decision responses than L1 non-cognate controls. In a study employing *non-cognate* translation pairs and a masked priming paradigm, Schoonbaert et al. (2009) demonstrated translation priming effects not only from L1 to L2 (*meisje-GIRL*), but also from L2 to L1 (*girl-MEISJE*). These studies suggest that lexical activation in bilingual memory operates in a parallel, language non-selective way, and that L1 processing can be influenced by the weaker L2, even when the task is completed exclusively in the L1 (Kroll and De Groot, 2009). Although cross-language influences have been reported in both directions, L1 typically has a higher impact on L2 processing than vice versa (Keatley et al., 1994; Jiang, 1999; Schoonbaert et al., 2009). Several cross-language priming studies have found strong priming from L1 to L2 and weaker or no priming from L2 to L1 (Gollan et al., 1997; Jiang, 1999; Finkbeiner et al., 2004; Schoonbaert et al., 2009).

The present study tests cross-linguistic influences in the processing of binomials (*knife and fork*) – a type of MWEs for which this issue has not yet been examined. We investigate whether L1 influences the processing of congruent L2 binomials and whether this influence extends to the processing of translated L1-only binomials (i.e., binomials which have not been previously seen in L2). Secondly, we test whether a bilingual’s L2 influences the processing of binomials in the L1 and, if so, whether or not this influence is equally strong. We thus explore cross-language influences in both directions in the processing of binomials, addressing an important gap in MWE processing literature.

## The Present Study

To investigate cross-language influences in the processing of binomials, three groups of participants, Chinese–English and English–Chinese bilinguals and English monolinguals, completed the same English lexical decision experiment with a binomial priming manipulation. Both groups of bilinguals completed the experiment in the L2 immersion context. This design allowed us to investigate how the three groups of participants processed three types of MWEs: congruent, English-only, and translated Chinese-only binomials. In the case of Chinese–English bilinguals, we tested the involvement of L1 in L2 MWE processing, while with English–Chinese bilinguals, we tested the involvement of L2 in L1 MWE processing. The monolingual group of participants served as a baseline group.

Binomials are three-word phrases that are realized in English as an *A and B* form, where a specific word order is preferred (*knife and fork* vs. *fork and knife*) (Benor and Levy, 2006; Carrol and Conklin, 2020). They are highly fixed, that is, the reversed form is rarely used (Carrol and Conklin, 2020). The relative frequency of “*A and B*” vis-à-vis the reversed form “*B and A*” is quite central to binomials, in that “*A and B*” is always more frequent than “*B and A.*” The experiment investigated whether the first two words of a binomial phrase facilitate lexical access to the final word of the phrase. The participants were briefly shown the first two words of a binomial and a control phrase (*knife + and* OR *spoon* + *and*) and then made lexical decisions on the final word (*fork*). We compared response times on the final words of binomials (*knife and fork*) and control items (*spoon and fork*). Shorter response times on the final word of the binomials compared to the controls (i.e., MWE priming) was taken as evidence that the binomial expressions were processed as highly familiar, conventional phrases. To test cross-language influences in the processing of binomials, we wanted to determine whether congruent binomials (i.e., English binomials whose Chinese translation equivalents are also binomials in Chinese) would be processed faster than English-only binomials (i.e., English binomials whose Chinese translation equivalents are not binomials in Chinese) by the bilinguals (but not by the monolinguals). This congruency effect is a prominent marker of cross-linguistic influences. We further sought to determine whether translated Chinese-only binomials (i.e., Chinese binomials whose English translation equivalents are not binomials) were processed faster than their control phrases, in order to better understand and interpret the mechanisms underpinning congruency effect in MWE processing.

The research questions we sought to answer are:

(1) Is CLI observed in the processing of congruent L2 binomials by Chinese–English bilinguals?
(2) Is CLI observed in the processing of congruent L1 binomials by English–Chinese bilinguals?
(3) Is CLI observed in the processing of translated Chinese-only binomials with Chinese–English or English–Chinese bilinguals?

We predict that congruent binomials should be processed faster than English-only ones for Chinese–English *and* English–Chinese bilinguals. This is predicted by both the L1 MWE activation and L2 MWE experience accounts, although their proposed mechanisms responsible for the congruency advantage are different. We also predict that cross-linguistic influences should be greater in the L1-L2 direction than those in the L2-L1 direction, based on the findings reported in bilingual studies (Keatley et al., 1994; Jiang, 1999; Schoonbaert et al., 2009). In other words, we hypothesize that cross-language influences in the L2-L1 direction may occur but are likely to be weaker than those in the L1-L2 direction. In addition, we predict faster processing of translated Chinese-only binomials vs. controls in Chinese-English bilinguals, if the L1 MWE activation account is supported, or no MWE priming effect if the L2 MWE experience account is supported. English–Chinese bilinguals should show the same pattern in the processing of translated Chinese-only binomials as Chinese–English bilinguals, if their Chinese language proficiency is sufficiently high. Finally, we predict that English–Chinese bilinguals may process L1 MWEs in a different way from English monolinguals when they are in an L2 immersion context, due to the need to inhibit interference from their L1, especially if their knowledge of L2 is comparatively weak. This prediction is based on studies on the influence of L2 immersion on L1 processing which have found that bilinguals immersed in an L2 environment show slower processing speed in L1 compared to those who have not experienced immersion (Linck et al., 2009; Baus et al., 2013; Morales et al., 2014).

## Materials and Methods

### Participants

Three groups of participants were recruited for the study: Chinese–English bilinguals (*n* = 52), English–Chinese bilinguals (*n* = 51), and English monolingual controls (*n* = 52). The number of participants was estimated based on a repeated measures design with the expected effect size being around *d* = 0.3 for the power of 80% (Brysbaert and Stevens, 2018). Each participant received $10 for their participation in the experiment. The study was conducted with the ethics approval from Victoria University of Wellington (VUW).

Chinese–English bilingual participants were undergraduate and postgraduate international students and young professionals studying or working at VUW. They completed a language background questionnaire before the experiment, in which they reported their English proficiency test score (International English Language Testing System [IELTS] or Test of English as a Foreign Language [TOEFL]), the number of years in an English-speaking country (average = 3.86 years, range: 0.5 – 17 years), and an estimate of their daily usage of English (average = 48%, range: 10% – 90%). Their mean IELTS score^2^ was 6.67 (range: 6 – 8; roughtly equivalent to the levels B2-C1 of the Common European Framework of Reference for Larson et al., 2014). They were thus regarded as advanced speakers of English as a second language.

English–Chinese bilingual participants were undergraduate and postgraduate students from Peking University and Tsinghua University, China. They were L1 English speakers who came to study Chinese or other subjects in Beijing as international students. They completed a language background questionnaire before the experiment, in which they reported their Chinese proficiency^3^ (self-reported), the number of years of exposure in China (average = 1.92 years, range: 0.6 – 8 years), and the estimation of their daily usage of Chinese (average = 37%, range: 5% – 90%). Twenty-two participants reported themselves as intermediate speakers of Chinese as a L2, and 29 participants as advanced speakers of Chinese as a L2.

English monolingual speakers were also undergraduate and postgraduate university students and young professionals, from VUW. They completed a language background questionnaire before the experiment to make sure they had no knowledge of Chinese. Table 1 summarizes all participants’ language proficiency characteristics.

**TABLE 1:** Means (standard deviations) of self-reported age, L2 proficiency levels, daily usage of L2, years of exposure to L2 in L2-speaking countries.

	Chinese–English (*N* = 52)	English–Chinese (*N* = 51)	English monolinguals (*N* = 52)
Age	28.46 (6.16)	22.88 (2.85)	23.85 (6.04)
English proficiency	Advanced	Native	Native
Chinese proficiency	Native	Intermediate+	0.00 (0.00)
Daily usage of L2	English: 48% (23%)	Chinese: 37% (22%)	N/A
Years of exposure to L2	3.86 (3.88)	1.92 (1.84)	N/A

### Materials

The critical materials consisted of 60 binomials and 60 control phrases. The binomials were of three types: (1) congruent binomials (e.g., *sun and moon*), (2) incongruent English-only binomials (e.g., *bread and butter*), and (3) translated Chinese-only binomials (e.g., *wisdom and strength*). Each binomial was paired with a control phrase. Control items were created by replacing the first word of the corresponding binomial with an alternative word that was semantically related to the final word of the binomial condition (e.g., *knife and fork* vs. *spoon and fork*). Binomials and their corresponding controls thus differed only in the first word. Control items formed semantically plausible low frequency phrases. This resulted in 120 experimental stimuli (60 binomials and 60 controls), see Supplementary Appendix 1. Examples of the materials for each condition are presented in Table 2.

**TABLE 2:** Example of stimulus materials for each condition.

Condition	Binomial	Control
Congruent	Sun and moon	Star and moon
English-only	Bread and butter	Toast and butter
Chinese-only	Wisdom and strength	Exercise and strength

#### The Binomials and Their Phrase Frequency

The three types of binomials were chosen using the following criteria. First, for congruent binomials, the frequency of the binomial was much higher than the frequency of the reversed form in English *and* Chinese. For example, the binomial, *sun and moon* (太阳和月亮, taiyang he yueliang), is much more frequent than the reversed form, *moon and sun* (月亮和太阳, yueliang he taiyang), in English and Chinese: 30.54 vs. 6.25 occurrences (per 100 million words) in the Corpus of Contemporary American English (COCA: 560 million words) (Davies, 2008), and 38.63 vs. 7.77 occurrences (per 100 million words) according to the corpus of Center for Chinese Linguistics Peking University^4^ (CCL: 437.5 million words, Zhan et al., 2003). It can thus be classified as a true binomial both in English and Chinese. Congruent binomials and their reversed forms differed in phrase frequency in English (binomials: mean = 69.54, *SD* = 90.89; reversed forms: mean = 8.63, *SD* = 9.46; *t* = 6.95, *p* < 0.0001) and Chinese (binomials: mean = 66.21, *SD* = 75.41; reversed forms: mean = 6.57, *SD* = 9.38; *t* = 6.97, *p* < 0.0001).

Second, for English-only binomials, the frequency of the binomial was higher than the frequency of the reversed form in *English* but not in Chinese. The combination was legal in Chinese, but there was no word order preference in terms of frequency of occurrence. For example, the English binomial *bread and butter* is more frequent than the reversed form *butter and bread* (71.79 vs. 2.32 occurrences in COCA). However, the Chinese translation equivalent for the binomial *bread and butter*, 面包和黄油 (mianbao he huangyou), is almost as frequent as that of the reversed form *butter and bread*, 黄油和面包 (huangyou he mianbao): 3.89 vs. 1.83 occurrences in CCL. It is therefore classified as an English-only binomial. English-only binomials differed from their reversed forms significantly in phrase frequency (binomials: mean = 106.90, *SD* = 217.56; reversed forms: mean = 6.28, *SD* = 10.10; *t* = 7.20, *p* < 0.0001), whereas their Chinese translation equivalents were as frequent as their reversed forms (binomials: mean = 2.07, *SD* = 2.11; reversed forms: mean = 1.12, *SD* = 1.10; *t* = 1.66, *p* = 0.11). Additionally, to ensure the difference in the processing of congruent and English-only binomials could be attributed to the difference in congruency rather than phrase frequency, we also matched congruent and English-only binomials for phrase frequency in English (congruent binomials: mean = 69.54, *SD* = 90.89; English-only binomials: mean = 106.90, *SD* = 217.56; *t* = –0.08, *p* = 0.94).

Third, for Chinese-only binomials, the frequency of the binomial had to be higher than the phrase frequency of the reversed form in *Chinese* but not English. That is, for Chinese-only binomials there was no word-order preference in English. For example, the Chinese binomial *智慧和力量* (zhihui he liliang, wisdom and strength) was much more frequent than the reversed form *力量和智慧* (liliang he zhihui, strength and wisdom): 132.34 vs. 22.4 occurrences in CCL. By contrast, the English translation equivalent for the binomial *智慧和力量*, *wisdom and strength*, was almost as frequent as that of the reversed form *力量和智慧*, *strength and wisdom*: 3.04 vs. 4.64 occurrences in COCA. It was thus regarded as a Chinese-only binomial. Chinese-only binomials and their reversed forms differed in phrase frequency in Chinese (binomials: mean = 213.17, *SD* = 353.71; reversed forms: mean = 7.09, *SD* = 11.72; *t* = 8.98, *p* < 0.0001, but not in English (binomials: mean = 1.97, *SD* = 2.12; reversed forms: mean = 1.53, *SD* = 1.60; *t* = 0.50, *p* = 0.62).

Unlike English binomials which have a fixed structure of *A and B*, Chinese binomials are more flexible in form, in that they can take the following three forms: *A and B*, *AB*, and *A_、_B* (e.g., *knife and fork*: 刀和叉，刀叉，刀、叉). This reflects the characteristics of Chinese language, which is a paratactic language, whereby connective elements are often optional or unnecessary (Li and Ho, 2016). For binomials in Chinese, the word order is the most important attribute (i.e., A precedes B, rather than B precedes A), while the coordinator is not necessary. Thus, when we identified the frequency of occurrence of a Chinese phrase in CCL, we extracted its frequency in the forms of *A and B*, *AB*, and *A_、_B*, and used the sum of their frequency as the frequency of occurrence of this phrase. The controls, however, always had a conjunction (e.g., *和* he: and) in the Chinese version. In addition, when we translated Chinese-only binomials to English, the addition of the conjunction ‘and’ was necessary to conform to the *A and B* structure of English binomials. This kind of variation in form due to language differences is often inevitable in cross-language studies (e.g., Carrol and Conklin, 2014; Carrol et al., 2016).

Most of the binomials used in our study are literal phrases. However, in the congruent category, two items have a figurative and a literal meaning (‘song and dance,’ ‘thick and thin’). In the English-only category, three items have both a figurative and a literal meaning (‘bread and butter,’ ‘sticks and stones,’ and ‘bed and breakfast’). Therefore, literality was comparable across the different lists of binomials.

#### Association Strength

Following Siyanova-Chanturia et al. (2011), the University of South Florida (USF) Free Association Norms database^5^ was used to match the constituents (i.e., the first content word and the second content word) of the binomials (*sun and moon*) and the control items (*star and moon*) in forward association strength (*sun-moon* vs. *star-moon*: 0.15 vs. 0.115). This was needed to ensure that any processing advantage for binomials over their corresponding controls was not due to the first word in the binomials (*sun*) being a better prime than the first word in the control items (*star*) for the same target (*moon*) (e.g., Siyanova-Chanturia et al., 2011). There was no significant difference in forward association strength between the components of congruent and English-only binomials and their corresponding controls (congruent condition, *t* = 1.41, *p* = 0.17; English-only condition, *t* = 1.75, *p* = 0.15). However, for binomials and their controls in the Chinese-only category, the association strength between their constituents was not attested in the USF norm database. This was expected, since the USF is based on English, while no comparable Chinese database exists for the Chinese language. We only included the items which existed in the database.

#### Word Length and Frequency of the First Content Word

The first words in the binomial and control conditions were matched for part of speech, word length, and frequency (where possible). There was no significant difference between the first words in the binomial and the control conditions for word length (congruent condition, *t* = –1.19, *p* = 0.24; English-only condition, *t* = –1.04, *p* = 0.28; Chinese-only condition, *t* = –0.07, *p* = 0.94). However, while the first words in congruent and Chinese-only binomials and their corresponding control phrases were matched in terms of lexical frequency (congruent condition, *t* = 1.24, *p* = 0.23; Chinese-only condition, *t* = 0.52, *p* = 0.61), the first words in English-only binomials and their matched controls could not be matched (*t* = 3.01, *p* = 0.005). It was impossible to create plausible control items matched in frequency as well as forward association strength. To partial out any possible effect of the first word’s lexical frequency, we added the frequency of the first word as a covariate in our initial statistical model. The properties of the experimental items are presented in Table 3.

**TABLE 3:** Means (standard deviations) of phrase frequency, word length and frequency of first word, and semantic association strength for the binomial and control conditions (counts based on occurrences per 100 million words).

	Congruent		English-only		Chinese-only	
	Binomial	Control	Binomial	Control	Binomial	Control
Phrase frequency (English corpus) (Chinese corpus)	69.54(90.89) 66.21(75.41)	0.95 (1.03) 0.55(1.21)	106.90(217.56) 2.07(2.11)	0.76(0.94) 0.13(0.23)	1.97(2.12) 213.17(353.71)	0.52(0.57) 0.56(1.28)
First word length	5.45 (1.70)	6.05 (1.70)	4.8 (1.28)	5.3 (1.56)	6.4 (2.28)	6.55(2.48)
First word frequency	7636.60 (7400.19)	15633.22 (46006.48)	8559.96 (8485.87)	4544.17 (7898.84)	5700.86 (5918.46)	5698.47 (9866.77)
Association strength	0.24 (0.22)	0.17 (0.15)	0.14 (0.17)	0.07 (0.09)	0.01(0.01)	0.01(0.03)^a^

*^a^For items in the Chinese-only category, we could only obtain the association strength between the constituents of six binomials and four control phrases. The values reported were based on these 10 items which exited in USF norm database.*

#### Fillers and Non-word Items

A set of fillers with the same syntactic structure as binomials was constructed to reduce the proportion of related prime-target pairs, following 1/5 ratio proposed by McNamara (2005). The fillers were grammatical but implausible (*business and soul*). Non-word items were created to make an equal number of word/non-word responses, with the syntactic structure of *word + and + non-word*. All non-words came from the ARC non-word database (Rastle et al., 2002). They conformed to the phonotactic rules of English and were matched with the other items for length (mean = 5.88 letters). Primes for the non-word targets were words that were not used in other conditions. See Supplementary Appendix 2 for fillers and non-word items used in the experiment.

### Design

A repeated-measures design was used, with each participant exposed to the critical items in both conditions; this allowed for a within-participant comparison of response times in the two experimental conditions, providing better control for individual differences (Millar, 2011). To control for the repetition effect, two counterbalanced presentation lists were constructed. Half of the critical targets per list were presented as binomials and half as control phrases. In addition, the numbers of stimuli of each congruency type in each list were also balanced, such that each list contained an equal number of congruent, English-only, and Chinese-only binomials. Participants were randomly assigned to one of the two groups in the order of their participation. Group 1 saw List A first and then List B, and for Group 2 the order was reversed. The same number of participants was assigned to each group.

### Procedure

The experiment was conducted in a laboratory using DMDX software (Forster and Forster, 2003). Participants first read instructions on the computer screen and then completed 20 practice trials. All items were presented in the middle of the screen in white lowercase letters in Courier New font, size 24 pt, over a black background. At the start of each trial, a fixation point (“+++++”) was presented in the middle of the screen for 500 ms. It was replaced with the first word prime (*knife* in “*knife and fork*”), which was displayed for 250 ms. After that, a blank screen was presented for 150 ms (inter-stimulus interval [ISI] = 150 ms). Then the second word prime “*and*” was displayed for 250 ms, followed by the same ISI (150 ms). Finally, the target appeared and remained on the screen until a response was made, or the item timed out at 3,000 ms. The procedure is summarized in the following diagram:


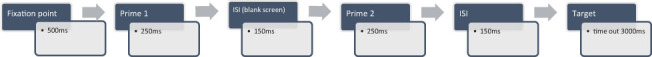


The items were presented in two counterbalanced blocks of 154 trials, with a self-paced break after Block 1. Within each block, the trial order was randomized for each participant. The whole experiment took approximately 20 minutes to complete.

## Analysis and Results

We analyzed accuracy and response latencies (RT). In the accuracy analysis, all responses were included. The mean response accuracy to non-word items was 95.94% for English monolinguals, 96.54% for English–Chinese bilinguals, and 82.18% for Chinese–English bilinguals. Accuracy was coded as a binary variable (1 – correct; 0 – incorrect). A generalized linear mixed-effects regression analysis was conducted to compare the accuracy between language groups. The likelihood ratio test indicated that there was a significant difference in response accuracy between the language groups (χ^2^ = 76.33, *p* < 0.0001). To further explore these differences, *post hoc* tests were run for the significant interactions, using emmeans() function in the R package emmeans (Lenth, 2019), with Bonferroni adjustments. The results showed that there was no significant difference in terms of accuracy for non-words between English monolinguals and English–Chinese bilinguals (*z* = –0.86, *p* = 0.39). However, Chinese-English bilinguals had lower accuracy for non-word trials than English monolinguals (*z* = 8.18, *p* < 0.0001) and English–Chinese bilinguals (*z* = 8.95, *p* < 0.0001). On word trials, the mean accuracy was 98.39% for English monolinguals, 97.68% for English–Chinese bilinguals, and 97.27% for Chinese-English bilinguals. Pairwise comparisons showed a significant difference in response accuracy between English monolinguals and Chinese–English bilinguals (*z* = 3.21, *p* = 0.004), but not between English monolinguals and English–Chinese bilinguals (*z* = 2.04, *p* = 0.12), or between Chinese–English and English–Chinese bilinguals (*z* = –1.17, *p* = 0.73). Importantly, within each language group, there was no significant difference in response accuracy between the binomial and control conditions for the three congruency types (i.e., congruent, English-only and Chinese-only). That is, there was no response accuracy priming for any of the three types of binomials in any language group.

For the RT analyses, the data for non-word and filler items were excluded from the analysis. We performed the analyses on RTs to 60 binomials (20 items for each congruency type: congruent, English-only, Chinese-only) and their corresponding controls (120 items in total). Incorrect responses were removed from the RT analysis, resulting in the loss of 1.12% data for English monolinguals, 2.31% data for Chinese–English bilinguals, and 1.98% data for English–Chinese bilinguals. Extreme values (RTs longer than 2000 ms or shorter than 250 ms) were also excluded (e.g., Sprenger et al., 2006; Matsuno, 2017), which resulted in the loss of 0.14% data for English monolinguals, 0.56% data for Chinese–English bilinguals, and 0.17% data for English-Chinese bilinguals.

English monolinguals overall responded faster than Chinese–English bilinguals (monolinguals: mean = 497 ms, *SD* = 133; bilinguals: mean = 655 ms; *SD* = 219). There was a difference of about 150 ms in RTs on the targets between English monolinguals and Chinese–English bilinguals, which was consistent with previous studies (e.g., Jiang, 1999). The mean RT to the target words for English–Chinese bilinguals was 512 ms (*SD* = 147), which was 15 ms slower than that for English monolinguals. Means of RTs by condition for three groups of participants is shown in Table 4.

**TABLE 4:** Descriptive statistics: mean response times in ms (standard deviations) and difference between mean response times to the binomial and control phrases for English monolinguals, Chinese–English, and English-Chinese bilinguals in each of the six experimental conditions.

		Binomial	Control	Difference
English monolinguals	Congruent	479.42 (125.88)	505.11 (134.61)	25.69
	English-only	471.07 (132.57)	492.68 (129.82)	21.63
	Chinese-only	519.12 (138.14)	513.56 (128.80)	–5.56
Chinese–English bilinguals	Congruent	637.84 (221.73)	657.86 (213.51)	20.02
	English-only	641.41 (204.58)	645.09 (205.91)	3.68
	Chinese-only	676.97 (241.64)	669.25 (222.02)	–7.72
English–Chinese bilinguals	Congruent English-only Chinese-only	495.43(128.65) 501.56(152.66) 528.79(158.83)	516.26(14.013) 504.96(141.42) 523.23(152.79)	20.83 3.4 –5.56

*Difference is calculated with mean RT to controls minus mean RT to binomials.*

Following Carrol and Conklin (2014) and Wolter and Yamashita (2015), reaction time data for each group were analyzed separately with linear mixed effects model using R (R Core Team, 2016), using *lme4* package (Bates et al., 2015), and *lmerTest* package (Kuznetsova et al., 2015). Following Brysbaert and Stevens (2018), RTs were inverse transformed (i.e., −1000/RT) to bring the data closer to normal distribution. Inverse-transformed RTs were normally distributed, with skewness of 0.07 and kurtosis of 2.88. Inverse-transformed RT to the final word of each phrase was used as the response variable.

For each group, the model fitting procedure started with the same maximal model with participants and items treated as random-effect factors. The following predictors were included: (1) item type (binomial vs. control), (2) congruency (congruent vs. English-only vs. Chinese-only), (3) English phrase frequency (counts based on occurrences per 100 million words, log transformed), (4) the frequency of the first content word of a phrase (counts based on occurrences per 100 million words, log transformed), and (5) forward association strength between the first word and the last word of a phrase (based on USF database, log transformed). Block order (order in which participants saw the two presentation lists: Order 1 vs. Order 2) and the trial number of the presentation of the phrase in the experiment (scaled) were considered as fixed effects to account for repetition priming and the longitudinal effect of the experimental task on the behavior of the participants. The model included the following interactions: (1) item type and congruency, (2) item type and phrase frequency, (3) item type and the frequency of the first content word, (4) congruency and phrase frequency, and (5) association strength and congruency. Starting with the maximal model, we used step() function in *lmerTest* to arrive at the best model fit. The initial model with random slopes failed to converge, so we did not include random slopes at this stage. After fitting the best model, we conducted a forward stepwise model selection to identify the appropriate random effects structure with random slopes, using Akaike information criterion (AIC) values.

In order to address the issue of the collinearity between phrase frequency and item type, we orthogonalized phrase frequency by fitting a linear model in which phrase frequency was predicted by item type, following Siyanova-Chanturia et al. (2011). The residuals of this model (EngPhrFreq.Residual) were then used as our predictor of phrase frequency, such that effects of item type were partialed out.

After identifying the best model with random slopes, we visually inspected a quantile–quantile plot of the model’s residuals and removed 2.5 SD from the residuals to satisfy the assumption of homoscedasticity and normal distribution (data loss: 1.83% for English monolinguals; 1.85% for Chinese–English bilinguals, 1.87% data for English–Chinese bilinguals). We refit the model with the new data. The results for the identified model are shown in Table 5 for English monolinguals, Table 6 for Chinese–English bilinguals, and Table 7 for English–Chinese bilinguals.

**TABLE 5:** Results of mixed model for English monolinguals.

Fixed effect	Estimate	Standard error	df	*t*	*p*
Intercept	–1.98	0.04	97.86	–45.14	<2.00e-16
ItemTypebinomial	–0.10	0.02	5563	–5.58	2.59e-08
Congruency (C-only)	0.03	0.03	82.24	0.11	0.92
Congruency (E-only)	–0.06	0.03	76.85	–1.93	0.05
EngPhrFreq.Resid	–0.01	0.01	1308	–1.12	0.26
AssoStrength.log	–0.37	0.07	628.9	–5.01	6.96e-07
TrialNum.sc	–0.05	0.01	50.84	–4.41	5.43e-05
BlockOrder2	–0.13	0.05	50.08	–2.66	0.01
ItemType (binomial) *Congruency (C-only)	0.08	0.03	2963	2.45	0.01
ItemType (binomial) *Congruency (E-only)	–0.01	0.02	5921	–0.46	0.65
ItemType (control) * Wrd1Freq.log.c	0.01	0.01	1386	2.25	0.02
ItemType (binomial) * Wrd1Freq.log.c	0.04	0.01	1546	4.87	1.26e-06
**Random effects**	**Variance**	***SD***			
Target	0.008	0.09			
Participant	0.03	0.18			
TrialNum.sc | Participant	0.005	0.07			
Residual	0.12	0.35			

*df, degrees of freedom; Intercept levels: ItemType, control; Congruency, Congruent. Marginal R^2^ = 0.08, Conditional R^2^ = 0.33.*

**TABLE 6:** Results of mixed model for Chinese–English bilinguals.

Fixed effect	Estimate	Standard error	df	*t*	*p*
Intercept	–1.61	0.05	118.70	–29.81	<2.00e-16
ItemTypebinomial	–0.05	0.02	159.50	–3.39	0.0009
Congruency (C-only)	0.01	0.06	61.70	0.09	0.93
Congruency (E-only)	–0.06	0.06	58.76	–1.01	0.32
AssoStrength.log	–0.18	0.08	139.03	–2.27	0.02
TrialNum.sc	–0.08	0.01	52.99	–7.53	6.45e-10
ItemType (binomial) *Congruency (C-only)	0.06	0.02	58.92	2.38	0.02
ItemType (binomial) *Congruency (E-only)	0.06	0.02	186.20	2.63	0.009
**Random effects**	**Variance**	***SD***			
Target	0.03	0.18			
EngPhrFreq.Resid | Target	0.001	0.03			
TrialNum.sc | Target	0.0001	0.01			
Participant	0.06	0.24			
TrialNum.sc | Participant	0.004	0.07			
Residual	0.08	0.28			

*df, degrees of freedom; Intercept levels: ItemType, control; Congruency, Congruent. Marginal R^2^ = 0.04, Conditional R^2^ = 0.57.*

**TABLE 7:** Results of mixed model for English–Chinese bilinguals.

Fixed effect	Estimate	Standard error	df	*t*	*p*
Intercept	–2.01	0.04	99.99	–48.47	<2.00e-16
ItemTypebinomial	–0.05	0.02	130.49	–2.72	0.007
Congruency (C-only)	–0.04	0.03	84.37	–1.36	0.18
Congruency (E-only)	–0.07	0.03	77.60	–2.59	0.01
EngPhrFreq.Resid	–0.03	0.01	177.49	–2.74	0.006
Wrd1Freq.log.c	0.01	0.01	40.61	0.77	0.44
AssoStrength.log	–0.25	0.08	161.17	–3.21	0.002
TrialNum.sc	–0.04	0.01	51.70	–3.51	0.0009
ItemType (binomial) *Congruency (C-only)	0.02	0.04	173.83	0.55	0.59
ItemType (binomial) *Congruency (E-only)	0.06	0.02	179.42	2.32	0.02
ItemTypebinomial:wrd1Freq.log.c	0.02	0.01	176.52	2.08	0.04
**Random effects**	**Variance**	***SD***			
Target	0.007	0.08			
TrialNum.sc | Target	0.0003	0.02			
Wrd1Freq.log.c	0.0009	0.03			
Participant	0.06	0.24			
TrialNum.sc | Participant	0.004	0.07			
Residual	0.11	0.33			

*df, degrees of freedom; Intercept levels: ItemType, control; Congruency, congruent. Marginal R^2^ = 0.02, Conditional R^2^ = 0.41.*

### Results for English Monolinguals

**FIGURE 1:**
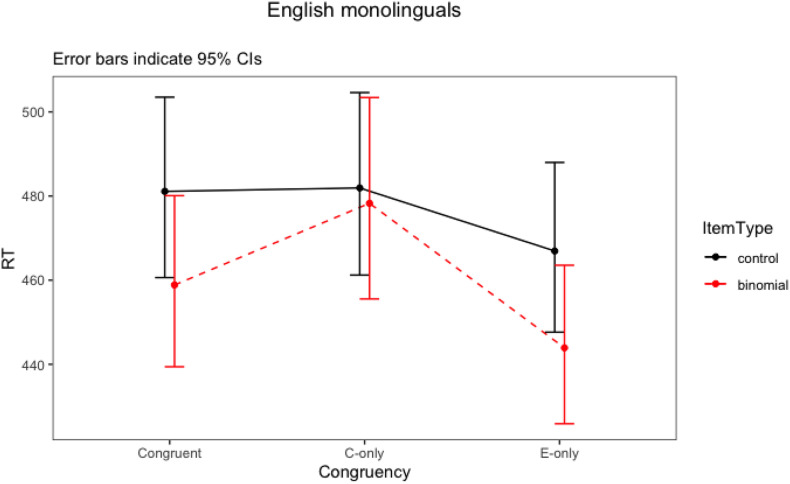
Interaction plot of Item type * Congruency for English monolinguals.

The final model for English monolinguals included two significant two-way interactions (item type × congruency, item type × Word 1 frequency). There were also statistically significant main effects of association strength, block order and trial number. The results suggested that more strongly associated phrases had overall shorter response latencies. Also, participants responded faster in Block 2 than in Block 1. They also went faster as the number of trials increased. The two-way interaction between item type and congruency (*F* = 3.82, *p* = 0.02) (Figure 1) showed that the English monolingual speakers processed congruent and English-only binomials significantly faster than their corresponding controls (i.e., priming effects are observed for congruent and English-only binomials), but there was no difference between Chinese-only binomials and their controls (i.e., no priming for Chinese-only). It also showed that priming effects for congruent and English-only binomials were comparable. To further explore these differences, *post hoc* tests were run for the significant interactions using emmeans() function in the R package emmeans (Lenth, 2019), with Bonferroni adjustments. The result is shown in Table 8.

**TABLE 8:** Results of *post hoc*, within-group tests of RTs for congruent, English-only, and Chinese-only items relative to the control items for English monolinguals.

Contrast	Group	Estimate	Standard error	*t*	*p*	ED (msec)
Ctrl-Cngr	ENS	0.101	0.018	5.573	<0.0001	22
Ctrl-E only	ENS	0.111	0.019	5.944	<0.0001	23
Ctrl-C only	ENS	0.016	0.025	0.645	0.99	4

*ED (estimated difference) is calculated with RTs to controls minus RTs to binomials.*

For the English monolinguals, priming effect was observed for the congruent (*t* = 5.73, *p* < 0.0001) and English-only conditions (*t* = 5.94, *p* < 0.0001). The mean RT^6^ to the binomials was 22 ms faster than RT to the control items (459 vs. 481 ms) in the congruent condition and 23 ms faster in the English-only condition (444 vs. 467 ms) (Table 8). We did not find priming for the Chinese-only condition (*t* < 1, *p* = 0.99), which confirmed that Chinese-only items were not processed by the English monolinguals as binomials.

### Results for Chinese–English Bilinguals

**FIGURE 2:**
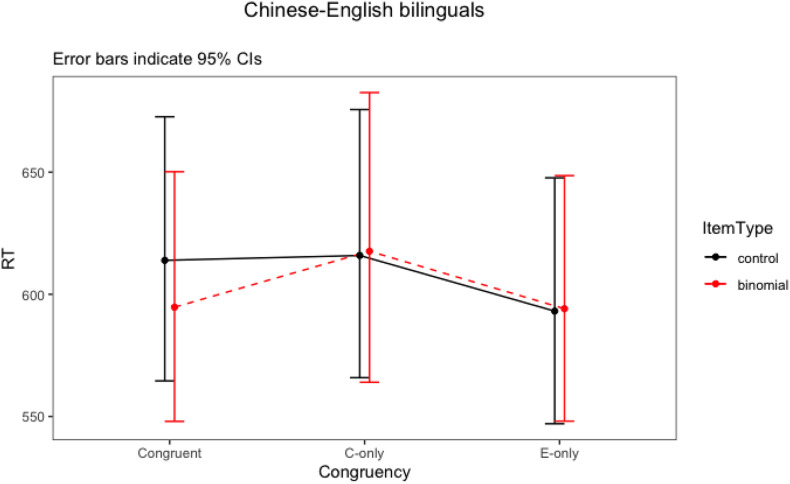
Interaction plot of Item type * Congruency for Chinese-English bilinguals.

The final model for Chinese–English bilinguals revealed a significant interaction between item type and congruency (*F* = 4.33, *p* = 0.016). There were also statistically significant main effects of association strength and trial number. Words within more strongly associated phrases had overall shorter response latencies. As the number of trials increased, the response time became faster. The two-way interaction (Figure 2) showed that the Chinese-English bilinguals processed congruent binomials significantly faster than the controls, but there was no difference between their processing of the English-only binomials vs. controls, nor any difference between Chinese-only binomials vs. controls. That is, only congruent binomials showed a priming effect. To further explore these differences, *post hoc* tests were run for the significant interaction using emmeans() function, with Bonferroni adjustments. The result is shown in Table 9.

**TABLE 9:** Results of *post hoc*, within-group tests of RTs for congruent, English-only, and Chinese-only items relative to the control items for Chinese–English bilinguals.

Contrast	Group	Estimate	Standard error	*t*	*p*	ED (msec)
Ctrl-Cngr	CE	0.053	0.016	3.388	0.013	19
Ctrl-E only	CE	–0.003	0.015	–0.187	0.99	–1
Ctrl-C only	CE	–0.005	0.018	–0.253	0.99	–2

*ED (estimated difference) is calculated with RTs to controls minus RTs to binomials.*

For Chinese–English bilinguals, the priming effect was only observed in the congruent condition (*t* = 3.39, *p* = 0.01), with RT to the terminal word in the binomials 19 ms faster than RT to the control items (595 vs. 614 ms, respectively). No priming effect was present for English-only binomials (*t* < 1, *p* = 0.99) nor Chinese-only (*t <* 1, *p* = 0.99) binomials: there was no difference between the binomials and the control items in English-only condition (594 vs. 593 ms) nor in the Chinese-only condition (618 vs. 616 ms). This suggests that only congruent expressions were processed as binomials, whereas English-only and Chinese-only items were not.

In sum, the relative processing advantage for congruent over English-only binomials compared to their corresponding controls was found for the Chinese–English participants, whereas for the monolingual participants no such difference was observed. In other words, the congruent binomials had a processing advantage over the English-only binomials for the Chinese–English bilinguals, even though the two types of binomials had been matched in phrase frequency.

### Results for English–Chinese Bilinguals

**FIGURE 3:**
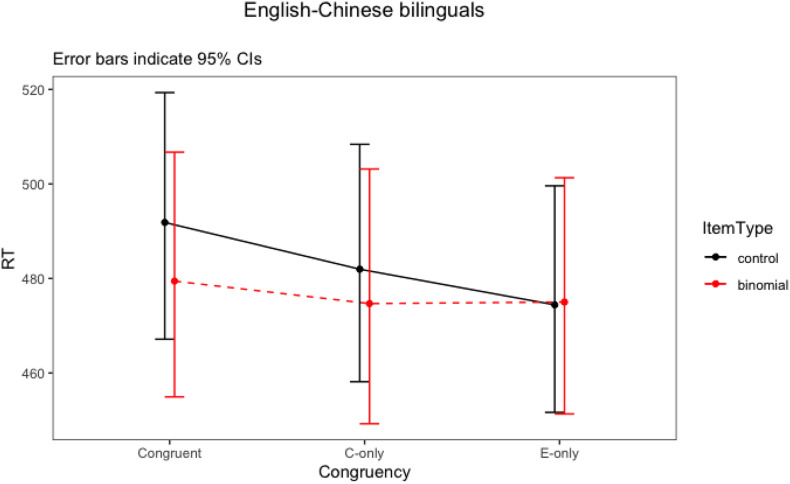
Interaction plot of Item type * Congruency for English-Chinese bilinguals.

The final model for English–Chinese bilinguals revealed a trend toward an interaction between item type and congruency (*F* = 2.73, *p* = 0.07). There were also statistically significant main effects of English phrase frequency, association strength and trial number. The model suggested that phrase frequency was always facilitative (led to lower overall RTs). Association strength was also facilitative whereby more strongly associated phrases led to lower overall RTs. Participants responded faster as the trial number increased. The two-way interaction (Figure 3) showed that the English-Chinese bilinguals processed congruent binomials somewhat faster than the controls, but there was no difference between their processing of the English-only binomials vs. controls, nor any difference between Chinese-only binomials vs. controls. To further explore these differences, *post hoc* tests were run for the significant interactions using emmeans() function, with Bonferroni adjustments. The result is shown in Table 10.

**TABLE 10:** Results of *post hoc*, within-group tests of RTs for congruent, English-only, and Chinese-only items relative to the control items for English–Chinese bilinguals.

Contrast	Group	Estimate	Standard error	*t*	*p*	ED (msec)
Ctrl-Cngr	EC	0.053	0.019	2.717	0.11	12
Ctrl-E only	EC	–0.003	0.019	–0.135	0.99	–1
Ctrl-C only	EC	0.032	0.027	1.19	0.99	7

*ED (estimated difference) is calculated with RTs to controls minus RTs to binomials.*

For English–Chinese bilinguals, there was a significant priming trend for congruent binomials. The magnitude of the priming effect was 12 ms (model estimate). However, it did not reach statistical significance, after a correction for multiple comparisons had been applied (*t* = 2.72, *p* = 0.11). There was therefore a *weak* priming effect for congruent binomials. In addition, no priming effects was observed in English-only (*t* < 1, *p* = 0.99) or Chinese-only (*t* = 1.19, *p* = 0.99) conditions.

## Discussion

The aim of the present study was to examine whether there were CLIs in the processing of MWEs, in the direction of L1-L2 as well as in the reverse direction, L2-L1. We focused on binomial expressions, that is, literal and compositional formulaic sequences, which have so far received little attention in cross-language processing research. To this end, we used a primed lexical decision task to examine the processing of three types of binomials vs. their corresponding matched infrequent controls: congruent English-Chinese (*sun and moon* vs. *star and moon*), English-only (*bread and butter* vs. *toast and butter*) and translated Chinese-only (*wisdom and strength* vs. *exercise and strength*). Three groups of participants, Chinese–English, English–Chinese bilinguals and English monolinguals, were tested.

### Cross-Language Influences From L1 to L2

English monolingual participants showed significant facilitation in the processing of the final word in English binomials compared to control phrases. The facilitation was irrespective of congruency; the magnitude of the priming effect was comparable in the congruent (22 ms) and English-only (23 ms) conditions. This offers further support to the tenet that binomials are processed differently from novel controls (Siyanova-Chanturia et al., 2011, 2017).

The Chinese–English bilinguals showed a priming effect (19 ms) in the processing of the terminal words of the congruent binomials compared to the control phrases, but no priming was observed for the English-only binomials (–1 ms). This indicates that there was a processing advantage for the congruent L2 binomials over the English-only binomials for the Chinese–English bilinguals (cf. RQ1). This result is in line with previous studies involving other types of MWEs. For example, in Wolter and Gyllstad (2011, 2013) and Carrol et al. (2016), bilinguals (but not monolinguals) showed a congruency advantage in the processing of congruent L2-L1 over L2-only idioms and collocations, respectively. Since congruent and English-only binomials were matched in English phrase frequency (*p* = 0.94) and did not show any difference in monolingual processing, we take the accelerated processing of congruent L2 binomials by Chinese–English bilinguals as evidence for the L1-L2 congruency effect. That is, CLI was observed in the processing of congruent L2 binomials by Chinese-English bilinguals.

#### L1 MWE Activation Account

As expected, the English native speakers showed no priming for the translated Chinese-only binomials over controls, since these word sequences were unknown to these participants. Importantly, the Chinese–English bilinguals did not show a significant priming effect for the translated Chinese-only binomials either (cf. RQ3), suggesting that translated Chinese-only binomials were not processed as binomials in the L2. This finding is inconsistent with the L1 MWE activation account of L2 processing, as shown in previous studies on the processing of translated L1-only *idioms* (e.g., Carrol and Conklin, 2014, 2017; Carrol et al., 2016). Carrol and Conklin reported a processing advantage for translated Chinese-only idioms over matched controls with Chinese–English bilinguals, in a primed lexical decision task (Carrol and Conklin, 2014) and in an eye-tracking study (Carrol and Conklin, 2017). Moreover, in an eye-tacking study with Swedish–English bilinguals, Carrol et al. (2016) replicated and extended this finding; they showed that translated Swedish-only idioms showed the same degree of processing advantage as congruent idioms, and that there was no more facilitation for congruent idioms than for Swedish-only ones due to their additional experience in the L2. This led them to conclude that, over and above direct experience in the L2, L1 MWE knowledge directly affects how translation equivalents are processed in the L2. This discrepancy between the present results and those of Carrol et al. (2016) may be due to the type of MWEs (i.e., idioms vs. binomials) and to the methodological differences between the studies, which will be further considered below.

Idioms are “strings of words whose figurative meaning does not necessarily derive from that of the constituent parts” (Cacciari, 2014, p. 267). That is, idioms have a figurative phrasal meaning and a literal meaning that reflects the meaning of their individual constituents. Thus, the processing advantage for idioms can come from form activation *and/or* from meaning activation (Carrol et al., 2016). Form activation refers to the recognition of specific word combinations presented in a particular order or configuration (i.e., lexical locus), while meaning activation refers to the understanding of the intended figurative phrasal meaning (i.e., conceptual locus) (Carrol et al., 2016). The robust advantage for translated L1-only idioms may come from meaning activation, although bilinguals may be unfamiliar with the form, when presented in the L2. For example, Beck and Weber (2016) found that translatable idioms (which have a matching concept *and* a word-for-word equivalent in L1) and untranslatable idioms (which have a matching concept *but* no translation equivalent in L1) produced comparable priming effect in proficient L1 German-L2 English bilinguals. This suggests that facilitation for the translated L1 idioms is likely to be driven by the conceptual overlap. In contrast, for literal MWEs (such as binomials), the processing advantage is likely due to form activation, i.e., based on the cooccurrence of the component word forms in a particular order.

Methodologically, the difference between the present study and studies on idioms is that the latter examined the processing of idioms in sentence contexts (Carrol et al., 2016; Carrol and Conklin, 2017), while the present study looked at the processing of binomials out of context. A biasing context greatly increases predictability in the processing of idioms (Titone and Connine, 1999; Cieślicka, 2013), which could have contributed to the translated L1-only idioms facilitation. Furthermore, Carrol and Conklin (2014, 2017) used very long idioms (e.g., *draw a snake and add* … *feet*), which may have allowed participants to actively anticipate the completion to a phrase (Carrol et al., 2016). Critically, most of the studies that found facilitation for translated L1-only idioms employed eye-tracking, while the present study employed a lexical decision task. Speeded primed lexical decisions rely on lexical level activation processes that are mostly automatic. Therefore, we chose the primed lexical decision paradigm to test for automatic cross-language activation.

#### L2 MWE Experience Account

Our findings are consistent with those reported in Wolter and Yamashita (2015, 2018). Wolter and Yamashita did not observe a processing advantage for translated Japanese-only collocations compared to non-collocational matched controls with Japanese-English bilinguals in two response-based tasks: a double lexical decision task (Wolter and Yamashita, 2015) and an acceptability judgment task (Wolter and Yamashita, 2018). Both in the present study and in Wolter and Yamashita (2015, 2018), translated L1-only MWEs were processed as unknown word combinations, suggesting that there was no automatic activation of known L1 MWEs in L2 processing.

The absence of priming for translated L1-only MWEs is predicted by the L2 MWE experience account which, similar to usage- and exemplar-based acquisition and processing accounts, assumes that frequency of encounters with and use of a lexical item (words, MWEs) determines quality of its mental representations and its ease of processing (Langacker, 2000; Bybee, 2006). A plethora of empirical studies have shown that frequency plays a key role in MWE processing (e.g., Arnon and Snider, 2010; Siyanova-Chanturia et al., 2011). It is argued that due to their frequency, MWEs are processed faster than matched novel phrases by L1 as well as L2 speakers (for a review, see Siyanova-Chanturia and van Lancker Sidtis, 2019). Since translated L1-only MWEs do not exist in the participants’ L2, they are unlikely to show a phrase frequency effect in the L2.

While the results for Chinese-English bilinguals suggest that known L1 MWEs are not automatically activated in the processing of the translated Chinese-only binomials, we are in no position to abandon the L1 MWE activation explanation entirely. For example, the original L1 MWEs may have been activated when the bilinguals read their L2 translation equivalents, but this activation may have been counteracted by the need to inhibit the non-target language (here, the participants’ L1, Chinese), since the task was completed entirely in the L2 (Green, 1998). Additionally, the Chinese–English bilinguals’ L1 may be inhibited, at the whole language level, in the context of their L2 immersion (Linck et al., 2009). Neurological studies have shown that competing information in the L1 needs to be suppressed to access information in an L2 (Abutalebi and Green, 2007; Pulido, 2021). Inhibiting L1 interference can improve L2 performance, in both immersion and non-immersion context (i.e., the L1 Regulation Hypothesis: Bogulski et al., 2019). In summary, the L1 inhibition necessitated by the experimental task and the country of residence contexts may have canceled out the possible activation of the L1 MWEs, resulting in no priming for translated Chinese-only binomials. In this case, the facilitation observed for the congruent L2 binomials could be due to their earlier acquisition by the bilinguals who, as a result, would have had a more extensive L2 processing experience with these binomials (as proposed in the L2 MWE experience account). This age of acquisition effect can also account for the congruency effect – the advantage in the processing of congruent over L2-only binomials. Since congruent and L2-only binomials were matched for L2 phrase frequency, there must be something other than L2 phrase frequency that contributed to the greater priming for congruent over L2-only MWEs. Age of acquisition of congruent L2 MWEs may well be such a factor. Wolter and colleagues (Wolter and Gyllstad, 2013; Wolter and Yamashita, 2015, 2018) argued that (1) congruent L2 MWEs are generally acquired earlier than incongruent L2-only MWEs, because acquisition is more straightforward when there is correspondence between the L1 and L2, and (2) earlier acquired congruent L2 MWEs are processed faster than later-acquired incongruent L2-only MWEs due to AoA effect. Because congruent MWEs share form (translation equivalents), structure (fixed word order) and referential meaning (same construct, e.g., *sun and moon*), they are more likely to be noticed in the L2 input and may be acquired faster (Yamashita and Jiang, 2010). One of potential mechanisms of the L1 transfer could be an initial strong declarative memory trace when encountering a congruent binomial in the L2 that exists in the learners’ L1. This initial declarative knowledge can facilitate the gradual acquisition of procedural knowledge from input, thus, the multiword sequence is acquired procedurally and may be processed faster and more automatically than L2-only MWEs (Ullman, 2014). Wolter and Gyllstad (2011) found that congruent collocations were processed faster than incongruent L2-only collocations with Swedish-English bilinguals in a primed lexical decision task. They interpreted the finding as evidence for L1 influence on the *development* of L2 collocational knowledge. Similarly, Yamashita and Jiang (2010) found that Japanese–English bilinguals made fewer errors on congruent collocations than incongruent L2-only collocations in a phrase-acceptability judgment task, irrespective of their L2 proficiency. This suggests that congruent L2 collocations show an acquisition advantage at the early stages of L2 learning. Incongruent L2-only MWEs, on the other hand, may need more repeated exposure to the L2 to be acquired. This account could also explain why no priming was observed for incongruent English-only binomials over the controls for the Chinese–English bilinguals in the present study. Similarly, in a lexical decision task, Wolter and Yamashita (2015) found that Japanese–English bilinguals did not produce accelerated processing for L2-only collocations either. It is thus plausible that the processing advantage for congruent over L2-only formulaic sequences is due to their age of acquisition. However, further empirical support is needed for the proposition that congruent MWEs are better noticed in the L2 input and are acquired earlier than incongruent L2-only MWEs (e.g., Arnon et al., 2017).

### Cross-Language Influences From L2 to L1

With respect to the performance of the English–Chinese bilinguals, our key findings were as follows. Unlike the English monolingual participants, the English–Chinese bilinguals did not show significant facilitation in the processing of the final word in the *English-only* binomial phrases (*bread and butter*) compared to the control phrases (*toast and butter*), but they showed a clear trend toward priming for the *congruent* binomials (*sun and moon*) compared to control phrases (*star and moon*). They processed congruent binomials quantitatively faster than their controls (mean difference = 20 ms, model estimate = 12 ms). However, after applying a correction for multiple comparison, the priming did not reach statistical significance (*p* = 0.11). Thus, compared with the Chinese-English bilinguals who showed significant priming in the processing of congruent binomials and a clear congruency advantage over English-only binomials, English–Chinese bilinguals showed only a weak congruency advantage (cf. RQ2). Finally, similar to the English monolinguals and Chinese-English bilinguals, the English-Chinese bilinguals showed no processing advantage for the translated Chinese-only binomials (*wisdom and strength*) over controls (cf. RQ3). We discuss each of these findings below.

#### The Inhibition of an L1

The finding that the English–Chinese bilinguals showed no processing advantage for English-only binomials over controls seems inconsistent with the literature on MWE processing in L1 speakers. It has been established that L1 speakers can recognize, read and respond to MWEs significantly faster than matched novel strings of language (Arnon and Snider, 2010; Durrant and Doherty, 2010; Vilkaite, 2016; Siyanova-Chanturia et al., 2017). In fact, we also observed a significant priming effect for English-only binomials for the English monolingual controls. What, then, might have caused the absence of priming for English-only binomials for the English–Chinese bilingual speakers, who performed the task in their native and dominant language?

One possibility is that the L1 of the English–Chinese bilinguals had been strongly inhibited in the L2 immersion environment (while studying Chinese in China). When they had to switch back to their strongly inhibited L1, for the purpose of completing the experiment, their L1 processing could have been impaired. The result that the mean RTs on L1 (English) lexical decisions were somewhat slower for the English-Chinese bilinguals than for the English monolinguals (512 ms vs. 497 ms; *p* < 0.0001) provides some evidence to support this conjecture. It has been shown that, after immersion in a foreign language, even just for a few months, bilinguals may experience delay when retrieving L1 words (Linck et al., 2009; Baus et al., 2013). Immersion is argued to enable bilinguals to attenuate the activity of the L1, thus better controlling L1 lexical competition and facilitating L2 learning (Linck et al., 2009). For instance, in a comprehension task (translation recognition), Linck et al. (2009) found that the immersed English-Spanish bilinguals showed no sensitivity to English distractors which had form overlap with the presented Spanish words (e.g., *cara-card*). The results were interpreted as evidence that immersed bilinguals suppress the visually presented distractors from intruding on their judgments, and that L1 was inhibited frequently during immersion to facilitate L2 learning. Recent evidence from classroom learning also indicates that the inhibition of L1 equivalents improves learning and retrieval of L2 MWEs in an L1-speaking environment (Pulido and Dussias, 2020; Pulido, 2021).

According to the Inhibitory Control model (Green, 1998), the non-target language is inhibited, preventing it from disrupting the selection of target language words. The amount of inhibition applied to the non-target language is proportional to the baseline strength of its activation. The more dominant the language, the stronger inhibition is needed. Since the L1 of an unbalanced bilingual is dominant, it is strongly suppressed whenever bilinguals need to use L2. As a result, the cost of reactivating L1 after using L2 is likely to be greater than a switch in the opposite direction (Mosca and de Bot, 2017; Wodneicka et al., 2020), having a greater effect on L1 performance. Numerous studies have shown that switching costs are larger for the stronger than for the weaker language (i.e., asymmetrical switching costs) (Meuter and Allport, 1999; Jackson et al., 2001; Macizo et al., 2012). For our unbalanced English-Chinese bilinguals, the L1 had to be strongly inhibited to enable them to use L2 in the immersion context. Switching back to their strongly suppressed L1, in order to perform an L1 lexical decision task, likely came at a cost. The absence of priming for English-only binomials in the English–Chinese group may have been a result of their weakened L1 performance.

The Chinese–English bilinguals who reported longer years of L2 exposure (3.9 vs. 1.9) and high proficiency in English may have been more balanced than the English–Chinese bilinguals and, therefore, may not have needed to inhibit their L1 as strongly. This would explain why their L1 could have been more readily activated during the processing of the congruent L2 binomials. This account is compatible with the extended Inhibitory Control model that is based on the language balance model (Wodneicka et al., 2020), which holds that the amount of inhibition applied to L1 during L2 use is related to the relative balance between the two languages. Studies have shown that when the two languages of a bilingual speaker are relatively balanced, the switching costs between languages becomes comparable, i.e., symmetrical switching costs (Christoffels et al., 2007; Schwieter and Sunderman, 2008; Declerck et al., 2013). In order to test this account, a follow-up study would need to compare the processing of L1 MWEs by bilinguals in an L2 immersion context and bilinguals in their L1 context. This would allow us to examine whether the impact of immersion on the L1 MWE processing is similar to that reported for single words (Linck et al., 2009; Morales et al., 2014).

#### L2 Influence on L1 MWE Processing

This difficulty of retrieving the dominant L1 in the L2 immersion context may also explain our findings for the processing of congruent L1 binomials by the English–Chinese bilinguals. The English–Chinese bilinguals showed some facilitation in the processing of the final word in congruent L1 binomials relative to control phrases, although the result was less robust than that observed for the Chinese–English bilinguals. This result is compatible with their performance on English-only binomials, suggesting attenuated L1 access due to L1 inhibition. We observed a clear trend toward priming for *congruent* L1 binomials that suggests possible activation of known corresponding L2 binomials. Since English-only and congruent L1 binomials were matched in L1 phrase frequency and our English monolingual controls showed comparable facilitation for both, activation of L2 binomial equivalents during the L1 task by the English–Chinese bilinguals seems to be the likely explanation of this priming trend for congruent L1 (but not English-only) binomials. This is evidence of cross-language influence in the L2-L1 direction in an entirely within-L1 task. This result is not unlike the findings of an automatic activation of single words in the weaker language in mixed stimulus lists (Dijkstra et al., 2000) and in L1-only lists (e.g., van Hell and Dijkstra, 2002). Our finding suggests that known L2 MWEs may be automatically activated in L1 processing, leading to the faster processing of MWEs that exist in both languages.

Finally, English–Chinese bilinguals showed no facilitation for translated Chinese-only binomials over controls. The same pattern of results was observed in English monolinguals and Chinese–English bilinguals. This indicates that there was no activation of translated Chinese-only MWEs (i.e., L2-only in the case of English–Chinese bilinguals). It is not surprising given that there was no activation for translated Chinese-only binomials over controls in Chinese–English bilinguals. In other words, the effects in the L2-L1 direction were less likely to take place when no such effects were observed in the L1-L2 direction, because CLI in the L1-L2 direction is normally stronger than in the opposite direction.

## Conclusion

In this study, we employed binomial expressions in order to examine crosslinguistic influences in the processing of MWEs in both directions. The results from Chinese-English bilinguals show that congruent L2 binomials showed greater priming effects than English-only binomials and that translated Chinese-only binomials showed no priming effect. We take these results as evidence that L1 influences the processing of binomials in the L2 and interpret them as supporting the L2 MWE experience account, according to which congruent MWEs should be processed faster than incongruent MWEs because they are noticed and acquired earlier due to the positive L1 transfer. English-Chinese bilinguals showed no priming for English-only binomials, but a clear priming trend for congruent binomials. These results support the view that L1 may be inhibited in L2 learning and immersion contexts and, thus, switching back to L1 may come at a cost. The results also support the view that crosslinguistic influence can occur from the non-dominant L2 to the dominant L1, even in an entirely within-L1 task. Thus, we conclude that crosslinguistic influences in the processing of binomials are bidirectional, although the influence in the direction of L1-L2 is stronger than in the reverse direction of L2-L1. This conclusion is in line with studies with bilingual word processing which suggest that crosslinguistic influences are bi-directional. The present study is the first study, to our knowledge, that investigated bi-directional cross-language influences in the processing of binomials – a less commonly studied type of MWEs.

## Data Availability Statement

The raw data supporting the conclusions of this article will be made available by the authors, without undue reservation.

## Ethics Statement

The studies involving human participants were reviewed and approved by Victoria University of Wellington, New Zealand. The participants provided their written informed consent to participate in this study.

## Author Contributions

LD, IE, and AS-C contributed to conception and design of the study. LD carried out the experiment and collected the data. LD and IE conducted the statistical analysis. LD wrote the first draft of the manuscript. IE and AS-C wrote sections of the manuscript. All the authors contributed to manuscript revision, read, and approved the submitted version.

## Conflict of Interest

The authors declare that the research was conducted in the absence of any commercial or financial relationships that could be construed as a potential conflict of interest.

## Footnotes

1 Most MWEs are high frequency word combinations. Some, however, exhibit low frequencies (e.g., idioms and proverbs, see Moon, 1998).

2 Among the 52 Chinese–English bilingual participants, there were 39 participants with IELTS scores, 4 participants with TOEFL scores, and 9 participants who had no English proficiency test record but took the University’s English Proficiency Program (EPP) and met their English proficiency requirement. TOEFL scores were converted to IELTS band scores for the ease of comparison based on the Comparison Table provided on the website: https://www.ets.org/toefl/institutions/scores/compare/.

3 Only nine participants had taken the standardized Chinese proficiency test called Hanyun Shuiping Kaoshi (HSK), which was not compulsory for their programs. Therefore, we used self-reported measures to assess their Chinese proficiency.

4 The CCL corpus contains 700 million Chinese characters. However, the character number is not equivalent to word number (e.g., 东西 dong xi, meaning ‘thing(s)’ in English, is considered as a two-character word). Following the Lancaster Corpus of Mandarin Chinese (McEnery and Xiao, 2004), we adopted a ratio of 1:1.6 between words and characters to calculate the number of words in CCL, which amounts to 437.5 million.

5 http://w3.usf.edu/FreeAssociation/

6 Here and the following reported in-text are model estimates. We report descriptive statistics calculated prior to the data analyses in Table 4.

## Supplementary Material

Supplementary Appendix 1 Binomials and control items used in the experiment.

Supplementary Appendix 2 Filler and non-word items used in the experiment.
