# Supplementary material for: Cross-Language Influences in the Processing of Multiword Expressions: From a First Language to Second and Back
Source: Front Psychol. 2021 Jun 24;12:666520. doi: 10.3389/fpsyg.2021.666520 (PMC8264060; doi:10.3389/fpsyg.2021.666520)
Supplement: Supplementary Appendix 1 — Binomials and control items used in the experiment. [file Table_1.docx]

**Supplementary Appendix 1: Binomials and control items used in the experiment**

**English binomials**

| **Congruent binomials** | **Controls** | **English-only binomials** | **Controls** |
| --- | --- | --- | --- |
| Love and hate | Like and hate | Salt and pepper | Spice and pepper |
| Push and pull | Drag and pull | Hands and knees | Arms and knees |
| Knife and fork | Spoon and fork | Hunting and fishing | Sailing and fishing |
| Sun and moon | Star and moon | Drugs and alcohol | Grain and alcohol |
| Army and navy | Sailors and navy | Bread and butter | Toast and butter |
| Birth and death | Cancer and death | Bed and breakfast | Supper and breakfast |
| Facts and figures | Shapes and figures | Soap and water | Boat and water |
| Height and weight | Diet and weight | Pride and joy | Fun and joy |
| Wind and rain | Sunshine and rain | Milk and honey | Oats and honey |
| Vitamins and minerals | Vegetables and minerals | Apples and oranges | Carrots and oranges |
| Newspapers and magazines | Articles and magazines | Arts and crafts | Hobbies and crafts |
| Costs and benefits | Welfare and benefits | Sticks and stones | Bricks and stones |
| Theory and practice | Skill and practice | Car and truck | Van and truck |
| Song and dance | Show and dance | Eyes and nose | Face and nose |
| Tables and chairs | Stools and chairs | Cheese and crackers | Nuts and crackers |
| Master and slave | Servant and slave | Cold and flu | Fever and flu |
| Attitudes and behaviours | Manners and behaviours | Marriage and divorce | Wedding and divorce |
| Thick and thin | Skinny and thin | Cream and sugar | Honey and sugar |
| Science and technology | Computer and technology | Cops and robbers | Criminals and robbers |
| Deaf and dumb | Smart and dumb | Meat and dairy | Butter and dairy |

**Chinese-only binomials**

| **Translated Chinese-only binomials** | **Controls** |
| --- | --- |
| Poor and underdeveloped | Old and underdeveloped |
| Diligent and brave | Faithful and brave |
| Pots and bowls | Jars and bowls |
| Fish and shrimp | Crab and shrimp |
| Flowers and applause | Success and applause |
| Workers and peasants | Landlords and peasants |
| Chickens and ducks | Swans and ducks |
| Greetings and wishes | Prayers and wishes |
| Pigs and dogs | Bears and dogs |
| Wine and meat | Beer and meat |
| Drought and flood | Earthquake and flood |
| Dragon and phoenix | Bird and phoenix |
| Talent and beauty | Confidence and beauty |
| Cold and fever | Cough and fever |
| Knife and sword | Bow and sword |
| Wisdom and strength | Exercise and strength |
| Agricultural and rural | Industrial and rural |
| Experience and lessons | Knowledge and lessons |
| Peace and unity | Harmony and unity |
| Identity and status | Reputation and status |
