# Supplementary material for: Cross-Language Influences in the Processing of Multiword Expressions: From a First Language to Second and Back
Source: Front Psychol. 2021 Jun 24;12:666520. doi: 10.3389/fpsyg.2021.666520 (PMC8264060; doi:10.3389/fpsyg.2021.666520)
Supplement: Supplementary Appendix 2 — Filler and non-word items used in the experiment. [file Table_2.docx]

**Supplementary Appendix 2: Filler and non-word items used in the experiment**

| Item type | Item | Prime 1 | Target |
| --- | --- | --- | --- |
| filler | sky and ocean | sky | ocean |
| filler | business and soul | business | soul |
| filler | floor and legs | floor | legs |
| filler | chapter and mouse | chapter | mouse |
| filler | family and pain | family | pain |
| filler | crime and root | crime | root |
| filler | angel and verse | angel | verse |
| filler | wealthy and well | wealthy | well |
| filler | aches and hook | aches | hook |
| filler | far and late | far | late |
| filler | alive and high | alive | high |
| filler | fire and steel | fire | steel |
| filler | flesh and yummy | flesh | yummy |
| filler | early and mixed | early | mixed |
| filler | seal and pleasure | seal | pleasure |
| filler | flight and writing | flight | writing |
| filler | mood and carpet | mood | carpet |
| filler | food and stance | food | stance |
| filler | forgive and match | forgive | match |
| filler | fruit and gentlemen | fruit | gentlemen |
| filler | good and down | good | down |
| filler | chunk and distance | chunk | distance |
| filler | hope and punishment | hope | punishment |
| filler | hot and clean | hot | clean |
| filler | income and dream | income | dream |
| filler | intents and devils | intents | devils |
| filler | iron and friends | iron | friends |
| filler | ladies and drinks | ladies | drinks |
| filler | lock and ceiling | lock | ceiling |
| filler | pen and blood | pen | blood |
| filler | check and forget | check | forget |
| filler | neat and bad | neat | bad |
| filler | lamp and plant | lamp | plant |
| filler | taxi and curtain | taxi | curtain |
| nonword | number and yops | number | yops |
| nonword | street and plepped | street | plepped |
| nonword | ability and byled | ability | byled |
| nonword | seek and broursed | seek | broursed |
| nonword | fear and rhins | fear | rhins |
| nonword | purpose and zoys | purpose | zoys |
| nonword | beat and meeze | beat | meeze |
| nonword | object and dwould | object | dwould |
| nonword | federal and wronts | federal | wronts |
| nonword | academic and glorned | academic | glorned |
| nonword | subject and ginth | subject | ginth |
| nonword | property and floaned | property | floaned |
| nonword | surface and blize | surface | blize |
| nonword | collection and strurd | collection | strurd |
| nonword | cover and rhonth | cover | rhonth |
| nonword | value and flirred | value | flirred |
| nonword | press and greemb | press | greemb |
| nonword | private and flars | private | flars |
| nonword | stuff and jinth | stuff | jinth |
| nonword | agent and ined | agent | ined |
| nonword | modern and migns | modern | migns |
| nonword | sleep and skrymbs | sleep | skrymbs |
| nonword | risk and swield | risk | swield |
| nonword | medical and sheed | medical | sheed |
| nonword | fight and shrows | fight | shrows |
| nonword | adult and brores | adult | brores |
| nonword | exist and snorpse | exist | snorpse |
| nonword | yard and krirk | yard | krirk |
| nonword | report and gigns | report | gigns |
| nonword | building and naphed | building | naphed |
| nonword | church and phleffed | church | phleffed |
| nonword | top and lunks | top | lunks |
| nonword | shoulder and thoaned | shoulder | thoaned |
| nonword | pattern and sprighs | pattern | sprighs |
| nonword | positive and phrarned | positive | phrarned |
| nonword | price and smapps | price | smapps |
| nonword | recent and ghouled | recent | ghouled |
| nonword | future and gints | future | gints |
| nonword | bank and breld | bank | breld |
| nonword | mention and frurze | mention | frurze |
| nonword | finger and bryled | finger | bryled |
| nonword | painting and droaled | painting | droaled |
| nonword | refer and jepped | refer | jepped |
| nonword | describe and neffed | describe | neffed |
| nonword | wrong and shronck | wrong | shronck |
| nonword | rest and stighed | rest | stighed |
| nonword | detail and jide | detail | jide |
| nonword | camp and warced | camp | warced |
| nonword | budget and gwownse | budget | gwownse |
| nonword | heart and jonde | heart | jonde |
| nonword | product and knamped | product | knamped |
| nonword | involve and theps | involve | theps |
| nonword | performance and spict | performance | spict |
| nonword | challenge and flaced | challenge | flaced |
| nonword | battle and gnouled | battle | gnouled |
| nonword | agreement and splode | agreement | splode |
| nonword | return and claste | return | claste |
| nonword | situation and cleas | situation | cleas |
| nonword | defense and drand | defense | drand |
| nonword | author and spind | author | spind |
| nonword | method and chaumb | method | chaumb |
| nonword | reality and phlassed | reality | phlassed |
| nonword | civil and hamps | civil | hamps |
| nonword | explain and sorld | explain | sorld |
| nonword | court and gnants | court | gnants |
| nonword | sport and vempt | sport | vempt |
| nonword | focus and gherck | focus | gherck |
| nonword | heat and shans | heat | shans |
| nonword | violence and shrond | violence | shrond |
| nonword | mouth and durze | mouth | durze |
| nonword | response and gynx | response | gynx |
| nonword | shot and prunned | shot | prunned |
| nonword | consumer and brins | consumer | brins |
| nonword | threat and gwacs | threat | gwacs |
| nonword | victim and mieze | victim | mieze |
| nonword | kitchen and theph | kitchen | theph |
| nonword | brain and saumths | brain | saumths |
| nonword | spirit and kugns | spirit | kugns |
| nonword | judge and twund | judge | twund |
| nonword | travel and franns | travel | franns |
| nonword | track and triend | track | triend |
| nonword | client and zours | client | zours |
| nonword | annual and ghekked | annual | ghekked |
| nonword | professor and keffed | professor | keffed |
| nonword | vote and zows | vote | zows |
| nonword | born and phlince | born | phlince |
| nonword | prevent and rhurned | prevent | rhurned |
| nonword | plane and blapse | plane | blapse |
| nonword | variety and knirnde | variety | knirnde |
| nonword | neck and grourn | neck | grourn |
| nonword | employee and yompt | employee | yompt |
| nonword | fan and puiced | fan | puiced |
| nonword | senior and pald | senior | pald |
| nonword | forest and zorled | forest | zorled |
| nonword | species and pripped | species | pripped |
| nonword | nuclear and snoursed | nuclear | snoursed |
| nonword | literature and juild | literature | juild |
| nonword | replace and moost | replace | moost |
| nonword | video and smord | video | smord |
| nonword | admit and foosed | admit | foosed |
| nonword | credit and cield | credit | cield |
| nonword | freedom and scroars | freedom | scroars |
| nonword | thigh and throns | thigh | throns |
| nonword | aid and wofts | aid | wofts |
| nonword | possibility and pharned | possibility | pharned |
| nonword | global and pleaned | global | pleaned |
| nonword | citizen and driend | citizen | driend |
| nonword | corner and phraft | corner | phraft |
| nonword | effective and thraned | effective | thraned |
| nonword | puzzle and ghossed | puzzle | ghossed |
| nonword | crowd and drix | crowd | drix |
| nonword | customer and drouled | customer | drouled |
| nonword | reform and slaphed | reform | slaphed |
| nonword | key and noiced | key | noiced |
| nonword | critical and wext | critical | wext |
| nonword | strike and knigned | strike | knigned |
| nonword | neutral and wrunned | neutral | wrunned |
| nonword | gather and snild | gather | snild |
| nonword | complain and zacks | complain | zacks |
| nonword | access and skoursed | access | skoursed |
| nonword | score and zaft | score | zaft |
| nonword | recall and eed | recall | eed |
| nonword | labour and shraud | labour | shraud |
| nonword | dilemma and prersed | dilemma | prersed |
| nonword | classroom and phrence | classroom | phrence |
| nonword | stretch and darred | stretch | darred |
| nonword | option and grauced | option | grauced |
| nonword | debate and prold | debate | prold |
| nonword | stare and wriced | stare | wriced |
| nonword | concept and saphed | concept | saphed |
| nonword | comprise and neud | comprise | neud |
| nonword | complex and hoosed | complex | hoosed |
| nonword | fashion and thrinth | fashion | thrinth |
| nonword | restaurant and laffed | restaurant | laffed |
| nonword | front and scincs | front | scincs |
| nonword | publish and prins | publish | prins |
| nonword | touch and tews | touch | tews |
| nonword | ministry and sciled | ministry | sciled |
| nonword | bridge and creened | bridge | creened |
| nonword | consequence and drinned | consequence | drinned |
| nonword | release and ghewed | release | ghewed |
| nonword | mission and friled | mission | friled |
| nonword | troop and clurs | troop | clurs |
| nonword | announce and bloist | announce | bloist |
| nonword | liberal and twauve | liberal | twauve |
| nonword | section and dwood | section | dwood |
| nonword | chief and thwield | chief | thwield |
| nonword | faculty and forld | faculty | forld |
| nonword | review and fimps | review | fimps |
| nonword | tea and hect | tea | hect |
| nonword | gender and thruys | gender | thruys |
| nonword | bond and pleasure | bond | pleasure |
| nonword | slow and rhardes | slow | rhardes |
| nonword | wheel and broughg | wheel | broughg |
